# Cytokinins in Dictyostelia – A Unique Model for Studying the Functions of Signaling Agents From Species to Kingdoms

**DOI:** 10.3389/fcell.2020.00511

**Published:** 2020-06-19

**Authors:** Megan M. Aoki, R. J. Neil Emery, Christophe Anjard, Craig R. Brunetti, Robert J. Huber

**Affiliations:** ^1^Department of Biology, Trent University, Peterborough, ON, Canada; ^2^Institut Lumière Matière, CNRS UMR 5306, Université Claude Bernard Lyon 1, Université de Lyon, Lyon, France

**Keywords:** cytokinins, Dictyostelia, *Dictyostelium discoideum*, cytokinin biosynthesis, cytokinin signaling, development

## Abstract

Cytokinins (CKs) are a diverse group of evolutionarily significant growth-regulating molecules. While the CK biosynthesis and signal transduction pathways are the most well-understood in plant systems, these molecules have been identified in all kingdoms of life. This review follows the recent discovery of an expanded CK profile in the social amoeba, *Dictyostelium discoideum*. A comprehensive review on the present knowledge of CK biosynthesis, signal transduction, and CK-small molecule interactions within members of Dictyostelia will be summarized. In doing so, the utility of social amoebae will be highlighted as a model system for studying the evolution of these hormone-like signaling agents, which will set the stage for future research in this area.

## Introduction

### Cytokinin Roles in Plants and Beyond

**FIGURE 1:**
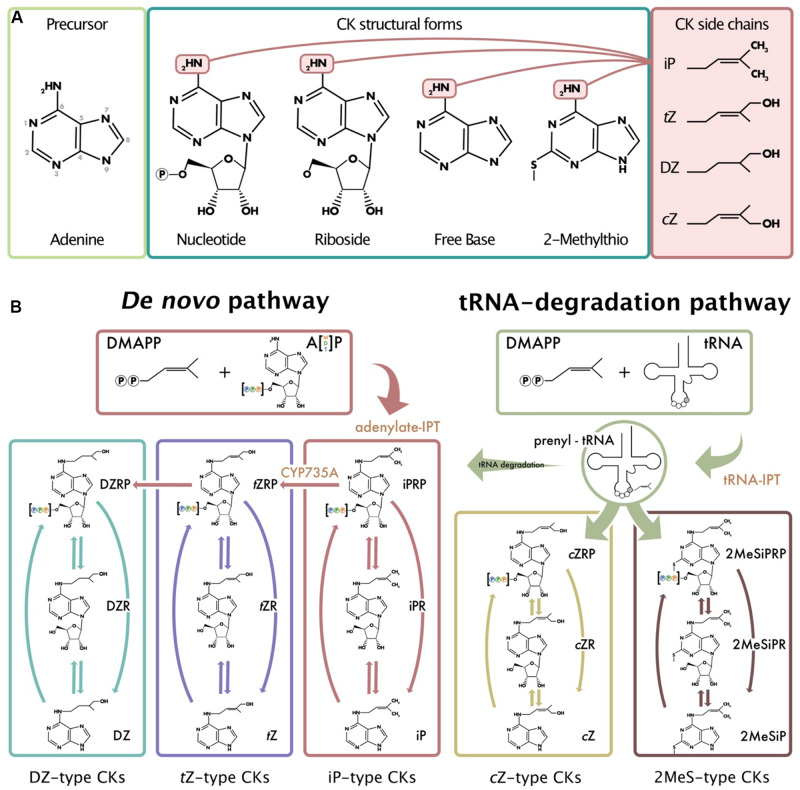
Common isoprenoid cytokinin (CK) structures and model of isoprenoid CK biosynthesis derived from plant, fungi, and bacterial models. **(A)** Depicts the numbering system used in CK nomenclature, the common CK structural forms, and the various isoprenoid side chains (iP, *t*Z, DZ, and *c*Z) that can be attached to the *N*^6^ position of the adenine. **(B)** Depicts the two different pathways from which isoprenoid CKs can be formed: the methylerythritol phosphate pathway (*de novo* pathway) and the mevalonate pathway (tRNA-degradation pathway). Arrows are representative of the various enzymes that convert between the different CK types and structural derivatives. Orange font indicates specific enzymes. The 2MeS-type CKs shown as a product of tRNA degradation all possess 2MeSiP-type side chains; however, 2MeS*t*Z- and 2MeS*c*Z-type are also possible. Note that not all CK structural forms and enzymes responsible for CK biosynthesis and metabolism are depicted (e.g., glucosides or cytokinin oxidase, etc.). Adapted from Kamada-Nobusada and Sakakibara (2009), Spíchal (2012), Morrison et al. (2015, 2017).

The cytokinins (CKs) encompass a group of evolutionarily significant molecules, most well known for their roles in signaling, where they orchestrate all levels of plant growth and development (Mok and Mok, 2001). Chemically, these molecules are *N*^6^ adenine derivatives, and they exhibit a broad phylogenetic occurrence from bacteria to humans sharing both common and diverging roles, such as growth promotion and virulence, among others (Figure 1A; Akiyoshi et al., 1984; Golovko et al., 2000; see reviews by Spíchal, 2012 and Kabbara et al., 2018). Recent genetic and molecular analyses have led to an explosion of research on the pleiotropic effects of these hormones in plant species (see reviews Kieber and Schaller, 2014; Durán-Medina et al., 2017). The early and simple documented roles of CKs at the single-cell level, such as the promotion of cell growth and differentiation, have now expanded to more complex roles involving whole-plant organization and beyond, such as: shoot initiation, leaf senescence, vascular and embryonic development, and nutrient uptake, among others (Kieber and Schaller, 2014). In concert, the elucidation of key elements in CK biosynthesis, perception, and signal transduction pathways have been pivotal to the understanding of these hormones on a molecular level.

In plants, biosynthesis of the most abundant CK type, the isoprenoid-type CKs, occurs through two different pathways: the methylerythritol phosphate pathway (MEP; *de novo* pathway) and the mevalonate pathway (MVA; tRNA-degradation pathway) (Figure 1B). Isoprenoid- (iP), *trans-*zeatin- (*t*Z), and dihydrozeatin-type CKs (DZ) are predominately derived from the *de novo* biosynthesis pathway through adenylate-isopentenyl transferases (IPTs) (Sakakibara, 2006). In contrast to the *de novo* pathway, the MVA or tRNA-degradation pathway is responsible for the production of *cis-*zeatin-type CKs (*c*Z) (Figure 1; Miyawaki et al., 2006). In fungi and mammals, there is evidence that iP- and methyl-thiolated-type (2MeS-) CKs are also derived from the tRNA degradation pathway (Morrison et al., 2017; Seegobin et al., 2018). In both pathways, IPTs facilitate N-prenylation of the adenosine molecule at the *N*^6^ terminus (Figure 1B) (Kakimoto, 2001; Takei et al., 2001; Sakakibara, 2006). When the isoprenoid donor dimethylallyl pyrophosphate (DMAPP) acts with IPT, isopentenyladenine-type (iP-type) CKs are formed. From iP-type CKs, other CKs can be generated (e.g., *t*Z, DZ) through modifications of the side chain. Specifically, adenylate-IPTs catalyze the transfer of the isoprenoid moiety to adenine through the rate limiting reaction to form iP nucleotides (either mono-, di-, or tri-phosphate; iPRPs). Therefore, the production of all other isoprenoid CK types is dependent upon the initial presence of IPT to facilitate iPRP production. In the case of tRNA-IPTs, prenylation occurs on tRNA molecules at position A37, and upon degradation, the tRNA-derived CKs contribute to the pool of unbound CKs in the organism (Gajdosová et al., 2011). From plant CK biosynthesis pathways, it is well-known that there are various CK types, and they are distinguished by their characteristic side-chain attachments at the *N*^6^ position of the adenine (e.g., iP or *t*Z) (Figure 1A; Kamada-Nobusada and Sakakibara, 2009). Within each CK-type, there are various structural derivatives or forms that exist which determine the level of biological activity within the organism, such as: free bases, ribosides and nucleotides, and conjugates with glucose, xylose, or amino acid residues (Figure 1A). Generally, the nucleotide forms are considered inactive precursors from which the more biologically active riboside and free base forms can be created.

Recently, an increasing amount of attention has been placed on uncovering the roles of CKs outside of the plant kingdom. In fact, these signaling molecules and or components of their biosynthesis pathways are present in organisms of all kingdoms. There is a possibility that CKs have roles in the growth and development of all organisms that produce them. Owing to their ubiquitous presence in all kingdoms, CKs are viewed as primary candidates for the study of interkingdom, hormone-like, signaling molecules (Kabbara et al., 2018). Recent evidence involving CKs opens many new doors for research beyond the plant kingdom. The human pathogen, *Mycobacterium tuberculosis*, secretes CKs that induce transcriptional changes affecting both the metabolome and staining properties of *M. tuberculosis* (Samanovic et al., 2018). These findings clearly demonstrate strong evidence of the involvement of CKs as signaling molecules on an interkingdom level. A phytopathogenic bacterial receptor was identified revealing a conserved mechanism by which bacteria can respond to CK in order to defend themselves against their host innate immune response (Wang et al., 2017; Chen et al., 2019). Moreover, a possible role for CKs in animal-microbiota relationships with implications for human health was explored in a recent review (Chanclud and Lacombe, 2017). In the apicomplexan parasite, *Toxoplasma gondii*, CKs regulate growth, the cell cycle, and apicoplast proliferation (which affects the viability of the parasite) (Andrabi et al., 2018). The thermophilic archaeon, *Sulfolobus islandicus* REY15A, was the first archaeal species identified to possess a CK-activating enzyme, lonely guy (LOG) (Mayaka et al., 2019). Furthermore, the detection of six additional CK forms, beyond the one previously identified (*N*^6^-isopentenyladenine-9-riboside), were reported in mammalian tissues (Seegobin et al., 2018). Among these various examples of CK production or activity in non-plant biota, there is a common theme of CKs exhibiting biological functions in many kingdoms of life. Exogenous application of CKs on mammalian cancer cell lines show that CKs have anticancer activity (Voller et al., 2010). Moreover, CKs affect growth and germination in the social amoeba, *Dictyostelium discoideum* and in the biotrophic fungus, *Claviceps purpurea* (Hinsch et al., 2015; Aoki et al., 2019). In the coming years, it is likely that we will continue to see increasing roles of CKs beyond the plant kingdom as research continues to use organisms from all kingdoms of life.

### The Dictyostelia

**FIGURE 2:**
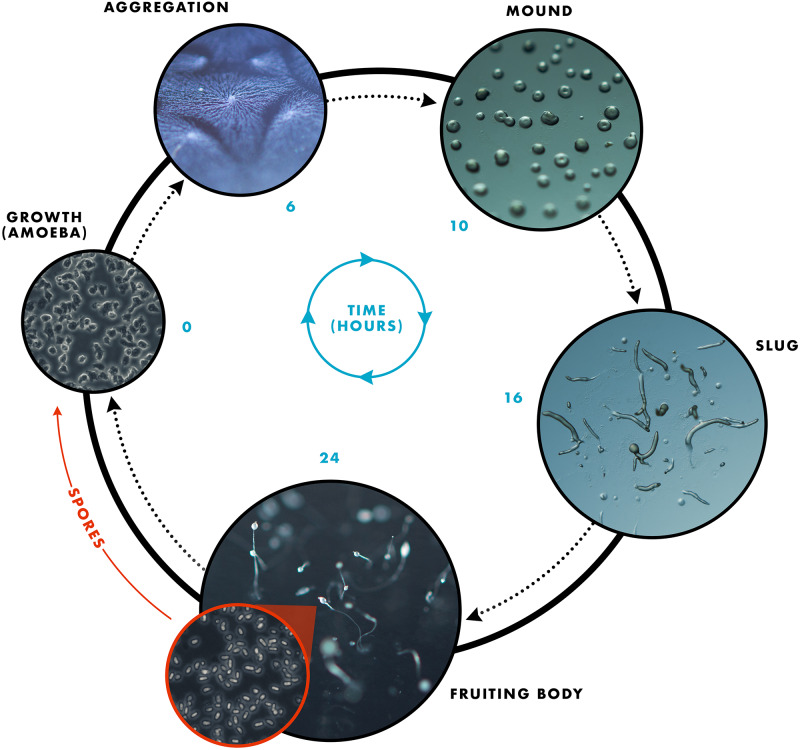
The *D. discoideum* life cycle showing the transition from a single-celled amoeba to a multicellular organism. Upon starvation, the developmental program is initiated in which single-celled amoeba aggregate toward a central location, as depicted in the aggregation image. The cells continue to aggregate to form the mound structure, which occurs after approximately 10 h starvation. Transition from the mound to the slug life cycle stage takes approximately 6–8 h. Finally, culmination generates a fruiting body consisting of a slender stalk and mass of spores that forms 24 h after the onset of starvation. Each spore gives rise to a single amoeba upon germination when food resources are available.

The Dictyostelids are soil-dwelling, amoeboid protozoans belonging to the Amoebozoa phylum (Raper, 1984; Romeralo et al., 2011). These eukaryotes are often referred to as social amoebae, owing to their unique life cycle, which consists of two mutually exclusive states: vegetative growth (single-celled amoebae) and development (multicellular organism) (Figure 2). Individual amoeboid cells grow asexually and divide mitotically, feeding upon soil bacteria and/or decaying leaf litter until resources are depleted. Starvation triggers the developmental program through the secretion of a chemical messenger, cAMP, which acts as a chemoattractant initiating the migration of neighboring amoebae (i.e., chemotaxis) to form an aggregate of cells (Konijn et al., 1967). These aggregates are collectively known as mounds. Tens of thousands of aggregated cells then undergo morphogenesis to form a multicellular pseudoplasmodium (slug), which migrates toward light and warmth (see review by Schaap, 2011). Cells within the slug terminally differentiate into either stalk or spores to form the final life cycle stage, a fruiting body, which consists of specialized stalk cells and a droplet of spores that sits atop the stalk (Schilde and Schaap, 2013; Loomis, 2014).

The most well-known organism of the Amoebozoa phylum is *D. discoideum*, hereafter referred to as *D. discoideum*. The genome of *D. discoideum* was the first free-living protozoan to be sequenced (Eichinger et al., 2005). Prior to sequencing, over five decades of intensive research on this social amoeba led to increased understanding of various cellular processes, such as chemotaxis and differentiation. Uniquely situated at the juncture of plants and animals, sharing many traits between the two kingdoms, the Dictyostelids offer a matchless platform to assess a wide variety of cellular and developmental processes. In light of the unique life cycle of Dictyostelid species, paired with its position in phylogeny and possession of CK biosynthetic and signal transduction components, this review will highlight how Dictyostelids can be used to study the role of CKs beyond the plant kingdom from an evolutionary perspective.

## Cytokinins in Dictyostelia

### Overview

The first articles published on the presence of CK in Dictyostelids involved the discovery of a novel CK in *D. discoideum*, known as discadenine (DA) (Obata et al., 1973; Tanaka et al., 1975; Abe et al., 1976). DA was discovered in the spore mass and was characterized as a potent inhibitor of spore germination. A notable discovery involving CKs in Dictyostelids by Taya et al. (1978a) revealed the existence of a CK biosynthetic pathway unrelated to the tRNA CK degradation pathway. Moreover, 5′-AMP was shown to be the acceptor molecule for the isopentenyl group forming the precursor molecule, isopentenyl adenine nucleotide (iPRP). Collectively, this early work in Dictyostelids laid the foundation for the CK biosynthesis pathway to be mapped in *Arabidopsis thaliana* (see reviews by Sakakibara, 2006; Kamada-Nobusada and Sakakibara, 2009). Following the initial discovery of an alternate biosynthetic pathway in Dictyostelids, the presence of *N^6^-*isopentenyladenine (iP) was identified at the start of fruiting body formation, also known as culmination, and iP was shown to be the precursor molecule to DA (Tanaka et al., 1978). Further analysis revealed a developmental regulation of CK in *D. discoideum*, as both iP and DA were detected following the onset of culmination (Ihara et al., 1980). This early evidence of the developmental role of CK in *D. discoideum* was later confirmed and expanded upon by Anjard and Loomis (2008). More recently, a comprehensive scan of 30 potential CKs in *D. discoideum* revealed that 6 different CKs are synthesized and secreted during growth, development, and germination (Aoki et al., 2019). Total levels of CK production were highest in the fruiting body and during germination, followed by aggregation and single-celled growth. Interestingly, iP-type CKs were the dominant CK forms during single-celled growth and aggregation, and DA was not present during these early life cycle stages. We detected high levels of DA following the onset of culmination, which is consistent with previous research (Ihara et al., 1980; Aoki et al., 2019). Exogenous application of iP affected single-celled growth by prolonging the stationary phase of cultures (Aoki et al., 2019). Together, these results indicate a greater role for CKs during the *D. discoideum* life cycle. The focus of this review will be on CK biosynthesis, metabolism, signal transduction, and CK-small molecule interactions in *D. discoideum*. We will also compare this information to what is known in other CK-producing organisms to provide an evolutionary context for CK function.

## Cytokinin Biosynthesis and Metabolism

### *iptA* – Adenylate Isopentenyltransferase

**FIGURE 3:**
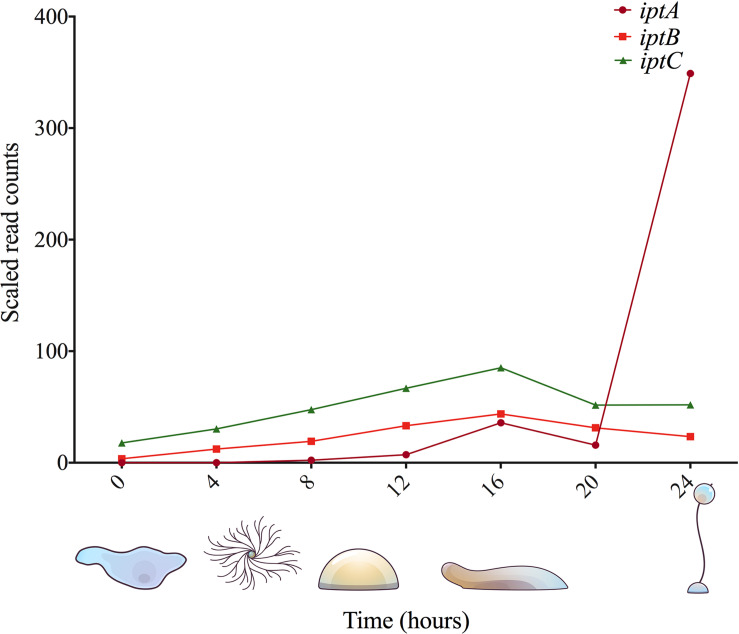
Gene expression analysis of known and putative CK biosynthesis genes in *D. discoideum.* RNA-Seq data was obtained from dictyExpress (www.dictyexpress.biolab.si) (Rot et al., 2009).

In *D. discoideum*, there are three identified IPT genes, only one of which has been functionally characterized (Anjard and Loomis, 2008). A phylogenic analysis of the corresponding proteins revealed that the *D. discoideum* genome encodes one adenylate-IPT and two tRNA-IPTs (Anjard and Loomis, 2008). The adenylate-IPT gene, denoted *iptA*, encodes a 283 amino acid, 32 kDa protein (IptA, DDB0233672) (Anjard and Loomis, 2008). IptA is a developmentally regulated protein, and its activity peaks during the late culmination stage of *D. discoideum* development (Figure 3; Ihara et al., 1980, 1984; Rot et al., 2009). Purification of IptA reveals that it is highly unstable and loses most of its activity after one day (Ihara et al., 1984). In terms of substrate specificity, 5′-AMP is the most effective substrate for IptA followed by ADP, which is 60–80% as effective. ATP, adenine, and adenosine are not substrates for IptA in *D. discoideum* (Ihara et al., 1984). The activity of *D. discoideum* IptA is dependent upon divalent metal cations (Zn^2+^, Mg^2+^, Mn^2+^), which is consistent with other prenyl-transfer reactions (Durbecq et al., 2001). The K_m_ values of *D. discoideum* IptA, under optimum conditions (pH 7.0, 1 mM Zn^2+^, and 25°C) for both 5′-AMP and isopentenylpyrophosphate are 1.0 × 10^–7^ M and 2.2 × 10^–6^ M, respectively (Ihara et al., 1984). While the role of IptA in *D. discoideum* is consistent with plants and other CK-producing biota, the aforementioned properties of the enzyme (K_m_, substrate specificity, etc.) are variable among CK-producing organisms, such as *A. thaliana*, and the plant-pathogenic bacterium, *Agrobacterium tumefaciens* (Kakimoto, 2001; Sugawara et al., 2008). For instance, the adenylate-IPT from *D. discoideum* and *A. tumefaciens* use AMP as the prenyl acceptor molecule, while *A. thaliana* adenylate-IPTs preferentially use ADP or ATP (Taya et al., 1978a; Kakimoto, 2001).

Disruption of *iptA*, through homologous recombination in the AX4 parental strain of *D. discoideum*, reduced total CK production and impaired spore viability (Anjard and Loomis, 2008). The rate of CK accumulation in *iptA*^–^ cells was 90% less than that observed in wild-type cells when developed from vegetative cells on filters over a 30-h period (CK production was assessed every 2 h starting from 20 to 30 h). Developing *iptA*^–^ cells with wild-type cells resulted in improved sporulation, indicating a non-cell autonomous phenotype (Anjard and Loomis, 2008). A threshold concentration of CK was calculated to determine the amount of endogenous CK necessary to fully induce spore formation, which was 10 nM. *iptA*^–^ cells took 30 h to reach this concentration, roughly 6–8 h longer than wild-type cells. As a result, these findings support the conclusion that CKs play a significant role in sporulation in *D. discoideum* (Anjard and Loomis, 2008).

**FIGURE 4:**
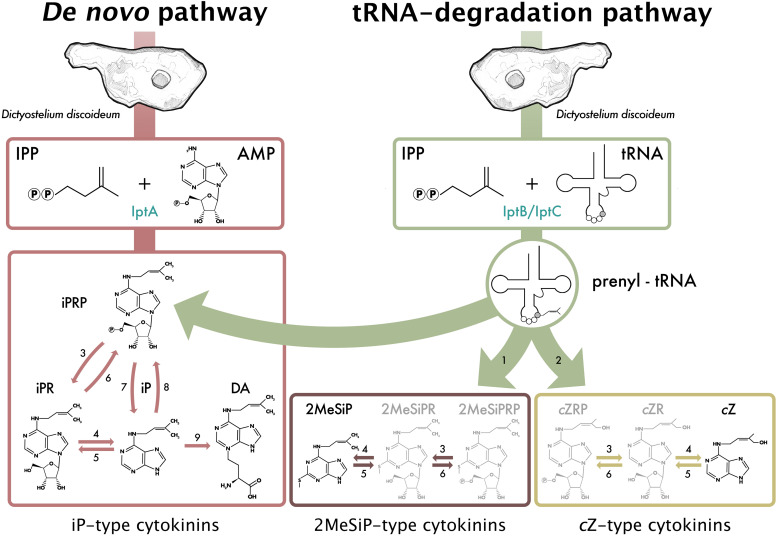
Proposed cytokinin (CK) biosynthesis pathway based on the CKs detected throughout all stages of the *D. discoideum* life cycle. Black CK structures denote CKs that have been previously detected in *D. discoideum*, and grayed CK structures are indicative of common CK forms detected in other CK-producing biosynthetic pathways. Numbers represent known or inferred enzymes from previous *D. discoideum*, plant, fungi, and bacteria studies and are as follows: 1. cdk5rap1-like ortholog (DDB_G0287079); 2. cis-hydroxylase; 3. 50-ribonucleotide phosphohydrolase; 4. adenosine nucleosidase; 5. purine nucleoside phosphorylase; 6. adenosine kinase; 7. CK phosphoribohydrolase (LOG-like ortholog, DDB_G0281309); 8. adenine phosphoribosyltransferase; 9. discadenine synthase (Taya et al., 1978a, b; Anjard and Loomis, 2008; Kamada-Nobusada and Sakakibara, 2009; Spíchal, 2012; Morrison et al., 2017; Nishii et al., 2018). Reproduced with permission from Aoki et al. (2019).

Although IptA is responsible for catalyzing the reaction for the synthesis of isopentenyl adenine-type (iP-type) CKs, organisms do not usually display a full complement of CK forms and this likely reflects the different pathways present and relative enzyme activities therein. Thus far, six CK forms have been identified in *D. discoideum*: *cis*-Zeatin (*c*Z), DA, iP, *N*^6^-isopentenyladenine-9-riboside (iPR), *N*^6^-isopentenyladenine-9-riboside-5′ phosphate (iPRP), and 2-methylthio-*N*^6^-isopentenyladenine (2MeSiP) (Figure 4; Abe et al., 1976; Tanaka et al., 1978; Taya et al., 1978a; Aoki et al., 2019). Of these identified CK forms, the most well-studied are iP and DA; DA will be discussed at greater lengths in a following section entitled discadenine sythase. The biosynthesis of iP-type CKs in *D. discoideum* appears to be similar to other CK-producing organisms, as the traditional CK forms of iP (nucleotide, riboside, and free base) have been identified throughout the life cycle (Aoki et al., 2019). Of the iP-type CKs identified in *D. discoideum*, the free base fraction, iP, is the most prevalent CK form detected (Anjard and Loomis, 2008; Aoki et al., 2019). Other than higher plants, only a handful of other CK-producing species contain adenylate-IPT genes: the plant pathogenic bacteria, *A. tumefaciens* (Akiyoshi et al., 1984) and *Rhodococcus fascians* (Crespi et al., 1992); the land moss, *Physcomitrella patens* (Lindner et al., 2014); the cyanobacterium, *Nostoc* sp. (Frébortová et al., 2015); and the social amoebae, *D. discoideum* and *D*ictyostelium *purpureum* (Anjard and Loomis, 2008; Sucgang et al., 2011^1^).

### *iptB* and *iptC* – Putative tRNA Isopentenyltransferases

In *D. discoideum*, there are two putative tRNA-IPTs: *iptB and iptC*. In the commonly used AX3 and AX4 strains of *D. discoideum*, there is a large duplication on chromosome 2, which affects *iptC* resulting in two copies of this gene, denoted *iptC-1* and *iptC-2* (Bloomfield et al., 2008). From phylogenic analyses, IptB and IptC cluster with tRNA-IPTs that are closely related to bacterial and eukaryotic tRNA-IPTs, respectively; however, there has been no functional characterization to date (Anjard and Loomis, 2008; Lindner et al., 2014; Nishii et al., 2018). Despite tRNA-IPTs being the most abundant IPT-type conserved across kingdoms of life, much less is known about it compared to adenylate-IPTs in terms of CK biosynthesis (Schäfer et al., 2015). *iptB* encodes a predicted 522 amino acid, 61 kDa protein (IptB, DDB0233673), and *iptC* encodes a predicted 413 amino acid, 48 kDa protein (IptC, DDB0233671). While these proteins have not been previously studied, it is likely that IptB and IptC contribute to the collective pool of CKs in *D. discoideum*, as the disruption of *iptA* leads to a drastic reduction of CK levels, but not a complete elimination of CK biosynthesis (Anjard and Loomis, 2008). In support of this idea, the recent CK profiles of *D. discoideum* follow a similar trend to what is found in CK-producing organisms with dominant tRNA-IPT CK pathways, possessing mostly iP-type CKs (Figure 4; Aoki et al., 2019). Therefore, the peak in expression at the 16-h stage of development (slug stage) could coincide with preparing the cells for the encapsulation of spores, where tRNA-bound CKs could be degraded to contribute to the pool of free CKs required for encapsulation at the 20-h time point (Figure 3^1^). In *D. discoideum*, there is little known about tRNA modification. Total tRNA transcript abundance does not increase during development, but tRNA modifications do (Palatnik et al., 1977; Rosengarten et al., 2017). The peak in expression of *iptB* and *iptC* throughout development aligns with these studies, which further support the putative roles of tRNA modification assigned to both of these gene products (Figure 3).

Beyond the role of CK biosynthesis, tRNA-IPTs are involved in many other functions, such as translation fidelity (in yeast and humans), *in vitro* growth and gene expression (in bacteria), and tRNA-gene mediated silencing (tgm) and drug resistance under environmental stress (in yeast), among others (Ericson and Bjork, 1986; Tolerico et al., 1999; Spinola et al., 2005; Suzuki et al., 2012; Pratt-Hyatt et al., 2013; Yu et al., 2017; see review by Dabravolski, 2020). Dabravolski (2020) summarizes that the roles of tRNA-IPTs can be broken up into different categories: (1) tRNA-isopentenylation-related, (2) tRNA-isopentenylation-unrelated, and (3) CK production upon tRNA degradation. Interestingly, a secondary zinc finger domain in addition to the IPP transferase domain (Pfam: IPPT) is required for the tgm role of certain tRNA-IPTs (Dabravolski, 2020 and references therein). IptB contains both of these domains, but IptC does not. Therefore, perhaps IptB has a secondary role involving tgm. With this in mind, more work with *iptB* and *iptC* knockout cell lines is necessary to determine the extent to which tRNA-IPTs contribute to CK production in *D. discoideum* and to other roles.

## Discadenine Synthase

**FIGURE 5:**
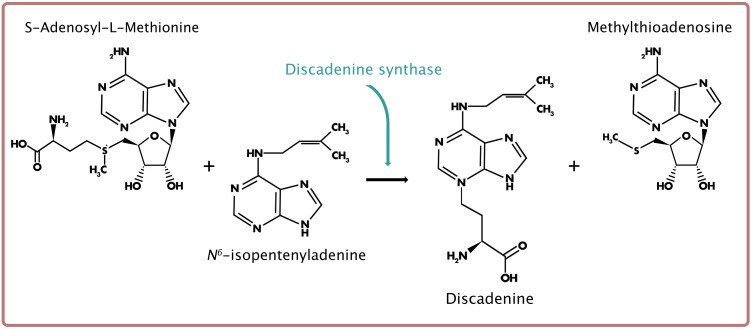
Reaction scheme for discadenine (DA) biosynthesis, derived from *N*^6^- isopentenyladenine (iP) and catalyzed by discadenine synthase.

Unlike both adenylate-IPTs and tRNA-IPTs, discadenine synthase (or synthetase) is unique to members of Dictyostelia (Taya et al., 1980; Abe et al., 1981). DA synthase is responsible for catalyzing the reaction that creates the novel CK and potent spore germination inhibitor, DA (Taya et al., 1978b; Ihara et al., 1980, 1986; Abe et al., 1981). Specifically, DA is synthesized through direct transfer of the 3-amino-3-carboxy-propyl moiety of S-adenosylmethionine (SAM) to iP (Figure 5; Taya et al., 1978b). While the gene encoding DA synthase remains unidentified, various properties of the protein have been studied in *D. discoideum* (Taya et al., 1978b; Ihara et al., 1980, 1986). Protein BLAST and HMMER searches in *D. discoideum* using known 3-amino-3-carboxypropyl transferases from other organisms, such as tRNA-uridine aminocarboxylpropyl transferase in *Escherichia coli*, to identify potential DA synthase gene candidates reveal no promising candidates.

The activity of DA synthase increases during *D. discoideum* development (Ihara et al., 1980). The protein is active earlier than IptA and continually increases in activity following aggregation, which includes a brief hold in activity after 16-h of development (slug stage), followed by a major peak at 26 h (post-fruiting body development) and a final smaller peak at 31 h. Considering that iP is the precursor to DA, it is puzzling that the activity of IptA peaks 10 h later than DA synthase (Ihara et al., 1980). The sharp rise in IptA activity near the onset of sporulation may indicate that large amounts of iP are rapidly converted to DA allowing for the precipitous encapsulation and dormancy of spores. In support of the incongruent enzymatic activities of IptA and DA synthase, similar findings were observed in *D. discoideum* between the developmentally regulated enzymes UDP-glucose epimerase and UDP-galactose polysaccharide transferase (Telser and Sussman, 1971).

The CK produced through DA synthase is unlike all other known naturally occurring CKs. Structurally, DA is an adenine derivative and is recognized as the first natural purine to possess an α-amino acid residue on the*N3* position of the adenine (Figure 5; Nomura et al., 1977). Unlike its precursor, iP, DA does not promote cell proliferation (at concentrations between 1 nM and 1 μM), nor does it prolong the stationary phase of single-celled growth (Aoki et al., 2019). However, when tested using the classical cytokinin assay (i.e., tobacco callus cell division bioassay), DA exhibits CK activity at concentrations tested between 0.5 and 16 μM (Nomura et al., 1977). More rigorous testing of DA as a CK was performed using several classical cytokinin and ligand-binding assays designed to assess CK activity in other organisms (Mik et al., 2017). The CK-like activity of DA was confirmed through the tobacco callus (cell division), *Amaranthus* (anthocyanin production), and detached wheat leaf (senescence) bioassays, and the activities were compared to that of a known potent, aromatic CK, benzyladenine (BA). The tobacco callus bioassay demonstrated most clearly the CK activity of DA, as the proliferation of CK-dependent callus cells was stimulated in a dose-dependent manner (at concentrations between 1 nM and 100 μM). Interestingly, the same concentration at which BA exerted cytotoxicity (10 μM) did not affect callus cell growth for cells treated with 10 μM DA. In fact, DA-treated callus cells continued to proliferate at this concentration. While *N3*-substituted CK derivatives (i.e., *N3-*methylated BA) have been shown to reduce CK activity, the *N3*-α-amino acid residue in DA was shown to decrease the cytotoxicity of the molecule compared to BA. As further confirmation of the CK-like activity, DA reduced the binding of isotopically labeled *t*Z in the *A. thaliana* receptors, AHK3 and CRE1/AHK4 (Mik et al., 2017). Of the two receptors, a higher affinity of DA occurred for CRE1/AHK4. Moreover, in a bacterial receptor assay, where the activation of the *A. thaliana* CK receptors results in the expression of the β-galactosidase reporter gene, DA elicited expression in both receptors, also in a dose-dependent manner (Mik et al., 2017). Therefore, DA possesses growth-promoting properties as a CK (in other organisms), as well as growth-inhibiting properties as an inhibitor of spore germination (in *D. discoideum*).

The proposed activities for DA may reflect a specific functionality within certain members of the *Dictyostelium* genus. A correlation between acrasins, or chemotactic agents, and spore germination inhibitors was noted between various Dictyostelid species (Taya et al., 1980; Abe et al., 1981). Of the 6 Dictyostelid species tested in these studies, DA and DA synthase were detected in only three species (namely *D. discoideum*, *D. purpureum*, and *Dictyostelium mucoroides*). These three species were the only species tested that use cAMP as their chemotactic agent for aggregation. Furthermore, inhibition of spore germination was achieved through treatment with 2 μM DA in these same three species, but not in the other three tested Dictyostelid species that utilize other chemotactic agents besides cAMP for aggregation (*Dictyostelium minutum*, *Dictyostelium lacteum*, and *Polysphondylium violaceum*). Based on these findings, it was postulated that spore germination inhibitors, including DA, may be both biochemically and evolutionarily linked with the chemotactic agent (Taya et al., 1980). This link with cAMP and DA will be discussed further in the CK-small molecule functional interactions section.

### LOG – CK Phosphoribohydrolase or Lonely Guy

**FIGURE 6:**
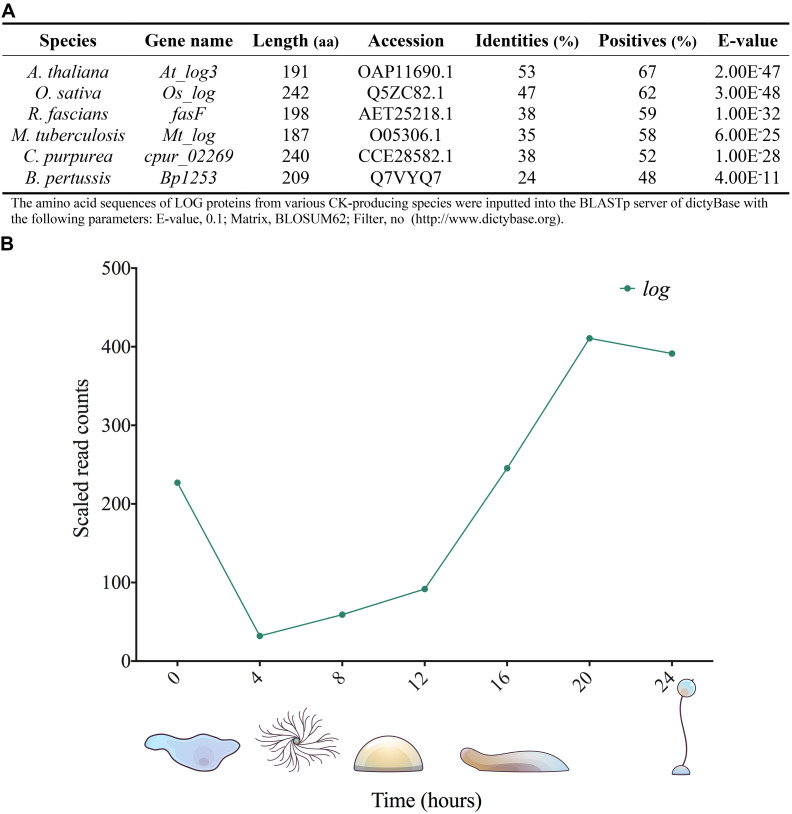
*Dictyostelium discoideum* has a putative *log* gene (DDB_G0281309). **(A)** presents LOG homologs from several organisms. Protein BLASTs were performed using the dictyBase BLAST server to assess sequence similarity of LOG homologs to the protein product of the putative *log* gene, DDB_G0281309 (http://www.dictybase.org). **(B)** Gene expression profile of the putative *log* gene using RNA-Seq data obtained from dictyExpress (www.dictyexpress.biolab.si) (Rot et al., 2009).

Cytokinin phosphoribohydrolase (LOG) is responsible for the direct conversion of CK nucleotides into their most biologically active forms, the free bases (Kurakawa et al., 2007). This enzyme was first discovered in rice (*Oryza sativa)* where a loss of function mutant resulted in decreased floral organs and flowers with only one stamen; accordingly, it was wryly named “lonely guy” (LOG). Since this discovery, LOG homologs have been characterized in other plant species as well as bacteria, fungi, and archaea (Kuroha et al., 2009; Hinsch et al., 2015; Samanovic and Darwin, 2015; Seo and Kim, 2017; Mayaka et al., 2019; Moramarco et al., 2019). A Protein BLAST search revealed a putative LOG homolog (DDB_G0281309) in *D. discoideum* that presents strong homology with known LOG genes from plants and bacteria (Figure 6A). Interestingly, there are two peaks in the expression of the putative *LOG* gene: during vegetative growth and late development, both of which coincide with a time when CK accumulation and biological effects have been observed in *D. discoideum* (Figure 6B; Anjard and Loomis, 2008; Rot et al., 2009; Aoki et al., 2019). *LOG* knockout and overexpression analyses in *A. thaliana* reveal pleiotropic effects on plant growth and development, and ultimately conclude that LOG plays a fundamental role in the regulation of CK across all developmental stages of *A. thaliana* (Kuroha et al., 2009). Considering the widespread occurrence of LOG genes among various kingdoms, it is highly likely this uncharacterized gene has phosphoribohydrolase activity in *D. discoideum*.

### CKX – Cytokinin Oxidase/Dehydrogenase

Cytokinin oxidase/dehydrogenase (CKX) inactivates CKs through oxidative cleavage of the *N*^6^ side chain from the adenine ring (Frébort et al., 2011). While CKX-activity has been identified primarily in plant species, similar enzymatic activities have been demonstrated in both *D. discoideum* and *Saccharomyces cerevisiae* (Van Kast and Laten, 1987; Armstrong and Firtel, 1989). An enzyme with CKX-like activity was assayed by Armstrong and Firtel (1989) throughout all stages of *D. discoideum* growth and development. There was a peak in enzyme activity from growth to aggregation and then a steady decline throughout the remainder of *D. discoideum* development. The purified enzyme catalyzed the cleavage of the *N*^6^ side chain from iP to adenine, and for this reason, was discussed as being similar to CKX in higher plants. However, Protein BLAST searches for both *D. discoideum* and *S. cerevisiae* do not reveal CKX-related sequences. Considering this point, Schmülling et al. (2003) speculates that CK breakdown via CKX is not conserved across all CK-producing organisms. In fact, the radish plant, *Raphanus sativus*, inactivates CKs solely through N-conjugation (Parker and Letham, 1973). The regulatory enzymes responsible for CK deactivation through N- or O-conjugation with a sugar moiety, commonly glucose, are known as uridine diphosphate glycosyltransferases (Šmehilová et al., 2016). A brief search on dictyBase revealed five characterized glucosyltransferases, four of which contained annotations for N-linked glucosylation. In our previous CK profiling experiments, we did not detect CK-glucosides at any stage of the *D. discoideum* life cycle; however, we did not search all possible conjugate alternatives (Aoki et al., 2019). It is likely that the observed CKX-like activity in *D. discoideum* occurs through a non-CK specific degrading enzyme, such as one of the mentioned *D. discoideum* glucosyltransferases. Further work is necessary to determine (1) if *D. discoideum* produces any CK-conjugates and (2) if so, what enzyme is responsible for CK deactivation.

## CK Secretion and Translocation

Like most hormones, CKs are synthesized intracellularly before being secreted into the extracellular space. Transporter proteins play a key role in the inter- and intracellular distribution of CKs. This topic has yet to be explored in *D. discoideum*, so we will draw on what is known in plant systems. In *A. thaliana*, the transmembrane ABC transporter, ABCG14, is responsible for the active transport of CKs from the roots to the shoots via the xylem (Ko et al., 2014; Zhang et al., 2014). This ATPase transporter localizes to the plasma membrane of root cells, and its inactivation prevents the translocation of CKs. Experiments using radioactively labeled CKs show that *At*ABCG14 acts as a CK exporter rather than an importer (Zhang et al., 2014). Potential regulation of CK secretion at the level of the ABC transporter is possible but has not been investigated. The *At*ABCG14 protein is characterized as a half-transporter, composed of both an ATPase binding domain and a transmembrane domain. To be functional, half-transporters must associate with another polypeptide containing both a binding domain and a transmembrane domain to form a homo- or heterodimer. Alternatively, genes encoding full ABCG transporters, with all four domains (two binding domains and two transmembrane domains) residing on a single polypeptide, are found in plants but not animals. Clear homologs of *At*ABCG14 are found across the plant kingdom and likely play a similar role in CK transport. The *D. discoideum* genome possesses a wide variety of ABC transporters encompassing 71 different genes, which includes 24 for the G family (8 half transporters and 16 full transporters) (Anjard et al., 2002; see also update in http://www.dictybase.org).

A sequence comparison shows that *At*ABCG14 presents strong homology with members of the *D. discoideum* ABCG family, especially *Dd*ABCG22. A null mutant of *Dd*ABCG22 was generated during a systematic study of ABC transporters (Miranda et al., 2013). This null strain presented delayed development and reduced spore viability – a phenotype that would be expected in the case of impaired CK secretion. However, the CK production of this strain was not evaluated. Furthermore, *Dd*ABCG22 was also found to influence vegetative cell dispersion during a screening for mutants reverting the dispersive phenotype of the ami8-mutant (Nagasaki and Uyeda, 2008). As CKs were recently shown to have a role during vegetative cell stage, such results are not unexpected (Aoki et al., 2019). Therefore, assessing CK production and secretion in the *Dd*ABCG22 null strain would give insight into whether this protein is involved in active transport of CKs or if its phenotype is unrelated to CK.

Two other protein families play a dominant role in CK transport in plants – the purine uptake permease (PUP) transporter family and the equilibrative nucleoside transporter (ENT) family. Certain members of the PUP transporter family allow for CK import into plant cells, specifically CK-nucleotides (Liu et al., 2019). The CK-specific PUP permeases appear to have evolved during terrestrial plant colonization between the bryophytes and the lycophytes from the pre-existing nucleotide sugar permease precursor (Jelesko, 2012). Thus, it is not surprising that homologs of PUP permease cannot be found in the *D. discoideum* genome. However, we cannot exclude the existence of a permease with similar function that would have evolved independently within Dictyostelids. The ENT family of transporters are also responsible for CK import into plant cells – specifically CK-ribosides (Liu et al., 2019). Out of the four ENT gene products in rice, one, *Os*ENT2 was shown to transport CK-ribosides, as well as adenosine (Hirose et al., 2005). *D. discoideum* possesses three uncharacterized ENT genes similar to *Os*ENT2 but also to other ENTs, making it impossible to predict their substrate specificity without further experimentation (Table 1).

**TABLE 1:** Sequence similarity of three uncharacterized equilibrative nucleoside transporter (ENT) proteins in *D. discoideum* to the known cytokinin transporter protein, *Os*ENT2, in *Oryza sativa*.

*D. discoideum*	*D. discoideum*	Length (aa)	Identity (%)	Positives (%)	E-value
Gene ID	Protein ID				
DDB_G0283439	DDB0273810	430	22	43	5.00E−13
DDB_G0281515	DDB0237816	522	21	41	8.00E−12
DDB_G0281513	DDB0237815	482	22	41	7.00E−11

*The amino acid sequence of *Os*ENT2 (Accession: BAG95361.1) was inputted into the BLASTp server of dictyBase with the following parameters: E-value, 0.1; Matrix, BLOSUM62; Filter, no (http://www.dictybase.org).*

## CK Signal Transduction

The canonical CK signal transduction pathway in plants involves a multi-step phosphorelay system that interacts through a complex form of the two-component signaling (TCS) pathway (Hwang and Sheen, 2001). TCS pathways are dominant in prokaryotes, especially bacteria, where they comprise the basic stimulus-response regulatory network allowing organisms to sense and respond to nearly all environmental stimuli (Stock et al., 2000). It is important to note that not all CK-producing organisms that respond to CK possess TCS elements. This is the case in several mammalian studies where CKs act as agonists to the adenosine receptors A_2A_ and A_3_ (Blad et al., 2011; Lee et al., 2012; Lappas, 2015). However, the remainder of this section will focus on components of the TCS CK signaling pathway, as are present in *D. discoideum.* Classically, the TCS pathway involves a histidine kinase (HK), which acts as the receptor, and a response regulator protein (RR), which, once activated, elicits a specific response through downstream effectors. This is slightly modified in land plants, where the CK signal transduction pathway involves a series of sequential phosphorylation events that alternate between His and Asp residues initiated by HKs, are perpetuated by histidine phosphotransfer proteins (HPs), and finished by RRs (Kieber and Schaller, 2018). A conserved extracellular loop of the HK transmembrane receptors belonging to the cyclase/histidine kinase-associated sensory extracellular (CHASE) domain-containing HK family is responsible for the initiation of CK signaling (Anantharaman and Aravind, 2001). Interestingly, the number of reported CK-producing organisms that possess conserved CHASE domains in recent years has expanded and will be described below (Kabbara et al., 2018).

### DhkA and AcgA

**FIGURE 7:**
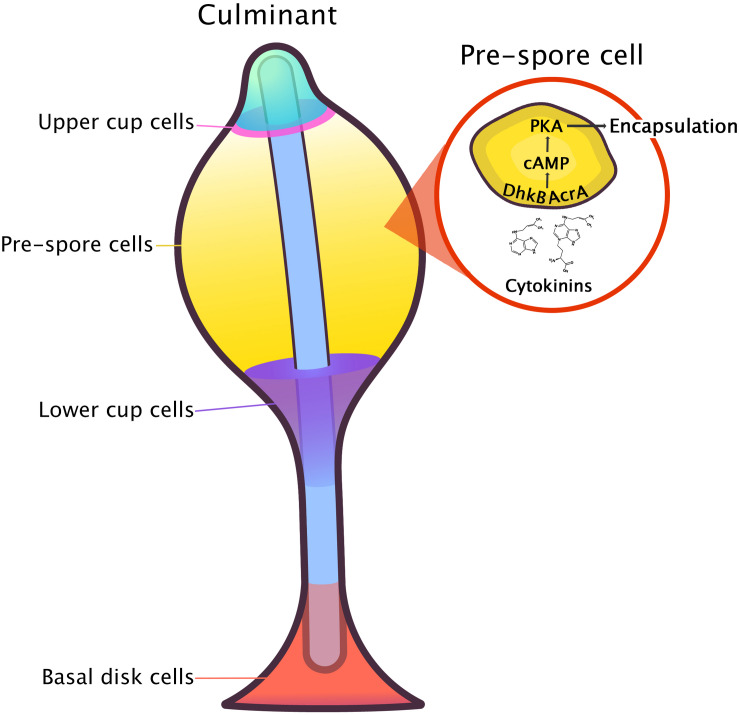
A model for the induction of sporulation by CK proposed by Anjard and Loomis (2008). Cytokinin is perceived by an unknown receptor and initiates a downstream phosphorelay through interaction of the histidine kinase, DhkB, and the adenylyl cyclase, AcrA This, in turn, leads to an increase in intracellular cAMP where the CK pathway and the SDF-2 signaling pathway converge at the level of PKA activation. An increase in PKA activity then leads to rapid encapsulation of spores.

In *D. discoideum*, there are two CHASE-domain containing proteins that are both involved in encystation and sporulation – the HK, DhkA, and the adenylyl cyclase of germination stage protein, AcgA (Alvarez-Curto et al., 2007; Anjard and Loomis, 2008). Neither of these proteins appear to be a CK receptor in *D. discoideum*, as null mutants for both proteins have a normal response to CK treatment (Anjard and Loomis, 2008). However, a *dhkA* and *acgA* double knockout has yet to be tested for definitive confirmation that neither of these proteins function as the CK receptor in *D. discoideum*. While CK was shown to act independently of DhkA, DhkA is still thought to regulate spore germination (Anjard and Loomis, 2008). In pre-spore cells, the peptide, spore differentiation factor 2 (SDF-2), binds to the CHASE domain in DhkA, which leads to the dephosphorylation of the cAMP phosphodiesterase, RegA, resulting in its inactivation. In this same time frame, the CK signaling pathway in *D. discoideum* (discussed below) converges to facilitate spore formation by inducing cAMP production (Figure 7; Anjard and Loomis, 2008).

### DhkB and AcrA

The CK signal transduction pathway leading to the induction of sporulation is dependent on the HK, DhkB, and the adenylate cyclase with response regulator domain, AcrA (Anjard and Loomis, 2008). *dhkB* encodes a 1,969 amino acid, 219 kDA protein (DhkB, DDB0215358), whereas *acrA* encodes a 2,123 amino acid, 243 kDA protein (AcrA, DDB0191294). *dhkB* is one of 13 functional HK genes in the *D. discoideum* genome that encodes a HK protein possessing several potential transmembrane domains and extracellular loops (Anjard and Loomis, 2003). Because of the large size of both of these transmembrane proteins, they were tested in a CK binding assay to determine if they were CK receptors (Anjard and Loomis, 2008). Wild-type cells (22-h) undergoing development were tested for their ability to bind ^3^H-labeled iP in the presence of 1 mM adenine to minimize non-specific background binding. Wild-type cells had a binding affinity for ^3^H-iP of 6 nM. Furthermore, specific binding of ^3^H-iP in vegetative and 22-h developed wild-type cells and 22-h developed *dhkB*^–^ and *acrA*^–^ cells was determined for each of the respective cell types. Developed 22-h wild-type, *dhkB*^–^, and *acrA*^–^ cells all bound similar levels of ^3^H-iP indicating that DhkB and AcrA are unlikely to be CK receptors. Vegetative wild-type cells bound 8-times less CK in comparison to the three tested developed cell types suggesting that the receptor is developmentally regulated. Zinda and Singleton (1998) reported that *dhkB*^–^ null cells develop into fruiting bodies within 22–24 h. However, precocious germination of spores quickly ensues as the mutant *dhkB*^–^ spores fail to respond to the spore germination inhibitor, DA. The germinated amoebae within the spore head quickly dehydrate owing to the high osmolarity maintained in the spore mass, and the majority of the cells are non-viable. *dhkB*^–^ cells with partially constitutive PKA activity (*dhkB*^–^/*K*), developed at low densities and failed to differentiate into spores when treated with DA or other CKs; however, they could differentiate when treated with the spore inducers SDF-1, SDF-2, and GABA at levels similar to those found in wild-type cells (Anjard and Loomis, 2008). AcrA carries two response regulatory regions, which were suggested as potential targets for the interaction between DhkB and AcrA (Anjard et al., 2001; see reviews by Kriebel and Parent, 2004 and Loomis, 2014). Like *dhkB*^–^ cells, *acrA*^–^ spores germinated precociously resulting in almost no viable cells (Söderbom et al., 1999). An abnormal phenotype was observed in *acrA*^–^ cells, in which the knockout strain formed fruiting bodies with abnormally thin stalks and glassy, translucent spore masses. Interestingly, this phenotype was also observed in the *dhkB*^–^ and *dhkA*^–^ knockout strains (Zinda and Singleton, 1998; Wang et al., 1999). Consistent with *dhkB*^–^ cells, *acrA*^–^ cells also failed to differentiate into spores following treatment with DA, iP, or *t*Z, but differentiated normally in response to other sporulation inducers (Anjard and Loomis, 2008).

### AcaA, RdeA, and RegA

Several other known proteins affecting *D. discoideum* development were studied to determine whether they were involved in the induction of sporulation by CKs (Anjard and Loomis, 2008). The adenylyl cyclase of aggregation protein, AcaA, was identified as a possible candidate for interaction with CK owing to its similarity to AcrA. *acaA*^–^ cells responded well to CKs, SDF-2, and GABA, indicating that the SDF-2 induction of spore encapsulation is not dependent upon a specific source of cAMP derived from this adenylyl cyclase (Anjard and Loomis, 2008). The rapid development protein, RdeA, and the previously mentioned cAMP phosphodiesterase, RegA, were tested to rule out any indirect effects of CK sporulation induction through stimulation of these proteins in the SDF-2 pathway. As mentioned earlier, spore induction through both the SDF-2 and CK signaling pathways occurs at precisely the same time during development. Previous work on spore induction pathways indicated that the peptide, SDF-2, generated from the Acyl-CoA binding protein, AcbA, acts on the receptor HK DhkA, which is present on both pre-spore and pre-stalk cells (Anjard and Loomis, 2005). The binding of SDF-2 to DhkA inhibits phosphorelay between RdeA and RegA. As such, the unphosphorylated response regulation region of RegA results in an accumulation of cAMP through AcrA, which increases the activity of PKA triggering rapid encapsulation of spores (Wang et al., 1999). Cells lacking RdeA and RegA responded to CK normally but were unable to respond to SDF-2 or GABA, thus leading to the conclusion that these proteins are not required for the induction of sporulation by CKs (Anjard and Loomis, 2008). While there have been no direct interactions observed between these three mentioned proteins and CKs, their combined roles lead to increased cAMP production, which is essential for encapsulation (Schaap and Schilde, 2018).

In summary, the signal transduction pathway for the induction of sporulation by CK is dependent upon DhkB and AcrA. CK signaling acts indirectly with the proteins involved in the SDF-2 signal transduction pathway, DhkA, RdeA, and RegA resulting in increased cAMP signaling. Furthermore, the other adenylyl cyclases, AcaA and AcgA, do not appear to directly interact with the CK pathway, unlike AcrA. However, there is an underlying connection with CK and cAMP in *D. discoideum* that will be discussed in the CK-small molecule functional interaction section. Potential cross-talk with CKs and other signaling pathways is likely and is common in other systems (e.g., CKs and auxin in plants); therefore, this would be an area of interest for future studies. Currently, there are no recognized receptors in *D. discoideum* that respond to CK, so further investigation is necessary to identify how CKs enact their effects. A candidate gene was found during systematic screening for developmental mutants after REMI mutagenesis. The mutant, DGG1110, presented a strikingly similar phenotype to the *acrA* null strain. Both strains develop normally but form spores that fail to remain dormant in the spore mass, leading to the death of the emerging amoebae. The gene inactivated in the DGG1110 strain (DDB_G0269128), encodes a predicted 544 amino acid protein with no clear homology to a known gene. However, this sequence presents a potential extracellular domain flanked by two transmembrane regions with a similar size and topology to the CHASE domain. While the homology with such domain is low, the mere presence of a potential extracellular loop in addition to the lack of response to CK in the null mutant make this gene a strong candidate for being the unidentified CK receptor in *D. discoideum*.

## Future Directions – CK-Small Molecule Functional Interactions

### CK-cAMP Interactions

cAMP is considered a universal second messenger controlling a wide variety of biological processes in all kingdoms of life (McDonough and Rodriguez, 2012). Among Dictyostelia, cAMP plays a dominant role in developmental signaling (Schaap, 2011). In the evolution of Dictyostelids, the roles of cAMP progressed from those involved in biological processes relating to stress to those regulating chemotactic aggregation, morphogenesis, and differentiation (Schaap, 2016). While the links between cAMP and the developmental program of *D. discoideum* have been extensively studied, intriguing suggestions of interactions between cAMP and CK have appeared over the last half century and remain largely unexplored.

Early studies on the role of CK in *D. discoideum* report an interesting correspondence between chemoattractants and spore germination inhibitors (Taya et al., 1980; Abe et al., 1981). As mentioned above, only the species that use cAMP as their acrasin also produce and respond to the spore germination inhibitor, DA. Moreover, during CK synthesis, the acceptor molecule of the isopentenyl side chain, 5′-AMP, which is catalyzed by IPT to create iP nucleotides, can be derived from cAMP through hydrolysis from cAMP phosphodiesterase (Taya et al., 1980). This evolutionary link between cAMP and CK among Dictyostelia was further confirmed through recent molecular phylogenetic analyses where all members of the most recently diverged species of Dictyostelia, group 4, use cAMP as their acrasin despite the use of various other chemoattractants among members belonging to the earlier diverged species of Dictyostelia in groups 1–3 (Schaap et al., 2006; Schaap, 2011). Therefore, it is likely that the earlier diverged species of Dictyostelia in groups 1–3 do not possess the enzyme to synthesize DA unlike the species belonging to group 4 who use cAMP as their chemoattractant.

Among other CK-producing organisms, links between cAMP and CK have been established. In *A. thaliana*, Plakidou-Dymock et al. (1998) identified seven potential seven transmembrane-spanning G-protein coupled receptors candidates. One of the candidates, *GCR1*, was isolated owing to its high similarity to other characterized 7-transmembrane proteins. Interestingly, the GCR1 protein is most closely related to the *D. discoideum* cAMP receptors, CarA-D. Transcriptome analysis of *GCR1* in *A. thaliana* revealed new evidence that this protein is involved in hormonal responses, including CK biosynthesis and response, among many other functions (Chakraborty et al., 2015). Therefore, it would be of high interest to study the Car receptors in *D. discoideum* as potential CK receptors. In *E. coli*, CKs have a growth-promoting effect by modulating the activity of enzymes responsible for cAMP biosynthesis and degradation (Judewicz et al., 1973; Coppola et al., 1976). A similar effect is seen in mammalian cells where exogenously added CKs act as competitive inhibitors of cAMP phosphodiesterase (Hecht et al., 1974). While a link between cAMP and CK is suggested through the reported studies, much remains to be understood between the functional interplay between these two molecules. Considering the dominant role cAMP plays throughout the *D. discoideum* life cycle, *D. discoideum* provides an excellent system to further unravel the interaction between CKs and cAMP.

### CK-NO Interactions

Like cAMP, nitric oxide (NO) is prevalent among all kingdoms and is one of the most common signaling molecules among living organisms (Schmidt and Walter, 1994; Lamattina et al., 2003; Moncada and Higgs, 2006). In *D. discoideum*, endogenous production of NO acts as a regulator of differentiation during development (Tao et al., 1997). NO-releasing compounds, such as sodium nitroprusside, inhibit *D. discoideum* aggregation by affecting the ability of cells to generate cAMP pulses (Tao et al., 1992, 1996). On the contrary, treatment with NO-scavengers, such as oxyhemoglobin (oxyHb), stimulates *D. discoideum* aggregation (Tao et al., 1997). Combining these respective results, a working model for the action of NO was proposed in *D. discoideum*: NO inhibits cAMP production in vegetative cells; upon starvation, the cells overcome the NO-inhibiting effects through cAMP pulses initiating the developmental program of *D. discoideum*. Total endogenous CK concentrations increase as cells transition from growth to aggregation (Aoki et al., 2019). This increase in endogenous CKs coincides with the decreases in NO that occurs during aggregation. Perhaps CKs have a secondary role in regulating NO levels in *D. discoideum*, specifically in the initiation of the developmental program.

Looking at other CK-producing organisms, it is clear that interactions between NO and CKs exist. A protective role of CKs against reactive nitrogen species was shown, in which CKs were shown to act as NO-scavengers (Liu et al., 2013). This same study reported that NO can chemically modify the CK structure to produce novel reaction products both *in vitro* and *in vivo*. Considering this evidence, future research looking into interactions between CK and NO in *D. discoideum* would be insightful to see if CK has a secondary role in scavenging NO during aggregation. Furthermore, the strong interactions between CK and NO, as demonstrated in plants and other CK-producing biota, could be used to elucidate candidate genes in *D. discoideum* involving NO synthesis and regulation (Husain et al., 2010; Feng et al., 2013; Liu et al., 2013; Samanovic and Darwin, 2015).

## Conclusion

The Dictyostelid system is unlike any other model organism utilized to assess the evolution of CKs as signaling agents among and between kingdoms of life. In fact, many new emerging areas of *D. discoideum* research encompass interactions with organisms in other kingdoms. These emerging areas include topics such as, *D. discoideum* as farmers of symbiotic bacteria, host defense against pathogenic bacteria and nematodes, and interactions with spore-dispersing organisms – all of which are influenced by interkingdom signaling, and perhaps CKs (Bozzaro and Eichinger, 2011; Brock et al., 2013; Smith et al., 2014; Novohradská et al., 2017). While there is accumulating evidence documenting the conservation of CK biosynthetic and signal transduction pathways among Dictyostelia species, much remains to be understood about the role of CK within this clade. Considering the widespread occurrence of CKs in both single-celled and multicellular organisms, Dictyostelids offer a unique opportunity to assess how CKs have evolved from roles at the cellular level to roles in controlling complex events during multicellular development. Furthermore, the dominant role that cAMP and NO play in various aspects of *D. discoideum* development expand the utility of this organism for studying not only the evolution of CKs beyond the plant kingdom, but also CK interactions with other signaling molecules. An additional promising small molecule to be investigated in the future for CK interactions is inorganic polyphosphate (poly P). Several studies highlight the drastic accumulation of poly P throughout the course of *D. discoideum* development, and *ppk1*^–^ cells have developmental defects in germination similar to those described for the CK-dependent *dhkB*^–^ and *acrA*^–^ null cells (Zhang et al., 2005; Livermore et al., 2016). Utilizing this model system, specific functions of CKs can be observed at the single cell level and beyond into multicellular organization and development, which may offer insight into how CKs have evolved as molecules, facilitating physiological interactions among and between various kingdoms.

## Author Contributions

MA and NE wrote the first draft of the manuscript. CA wrote sections of the manuscript. All authors contributed to manuscript revision, read and approved the submitted version.

## Conflict of Interest

The authors declare that the research was conducted in the absence of any commercial or financial relationships that could be construed as a potential conflict of interest.

## Footnotes

1 http://www.dictybase.org
